# Clock genes regulate skeletal muscle energy metabolism through NAMPT/NAD^+^/SIRT1 following heavy-load exercise

**DOI:** 10.1152/ajpregu.00261.2022

**Published:** 2023-08-07

**Authors:** Yu Xia, Binyu Yao, Zeting Fu, Lunyu Li, Songlin Jin, Bo Qu, Ying Huang, Haili Ding

**Affiliations:** ^1^Institute of Sports Medicine and Health, Chengdu Sport University, Chengdu, China; ^2^College of Physical Education and Health, Geely University of China, Chengdu, China

**Keywords:** clock genes, energy metabolism, heavy-load exercise

## Abstract

The biological clock is an invisible “clock” in the organism, which can regulate behavior, physiology, and biochemical reactions. However, the relationship between clock genes and energy metabolism in postexercise skeletal muscle is not well known. The purpose of this study was to determine the mechanisms through which peripheral clock genes regulate energy metabolism in skeletal muscle. We analyzed the rhythm of mRNA expression of the clock genes *Bmal1* and *Clock* in skeletal muscle following heavy-load exercise and measured related indicators of mitochondrial structure and function. We obtained the following experimental results. First, heavy-load exercise induced loss of circadian rhythm of *Bmal1* between ZT0 and ZT24, and the circadian rhythm of *Clock* was not restored between ZT0 and ZT72. Second, analysis of mitochondrial morphology in *group E* showed abnormal swelling and ridge structure damage at ZT0, which recovered somewhat at ZT24 and ZT48, and the damage had essentially disappeared by ZT72. Third, the expression of NAMPT/NAD^+^/SIRT1 signaling axis proteins in *group E* was abnormal at ZT0, the content of NAMPT and the activity of SIRT1 significantly increased, and the content of NAD^+^ significantly decreased. Fourth, the expression of BMAL1 and PGC-1α in *group E* significantly increased, whereas the ATP and ADP content, as well as the activities of COXII and COXIV, were significantly changed. Finally, the colocalization of BMAL1 and SIRT1 in *group E* was significantly upregulated at ZT0. These results suggest that the skeletal muscle clock gene *Bmal1* may regulate the energy metabolism level of skeletal muscle after exercise through the NAMPT/NAD^+^/SIRT1 signaling pathway.

## INTRODUCTION

The generation of biological rhythms is related to the rotation of the earth and the period of revolution around the sun. Biological rhythm encompasses regular periodic changes in physiological activities and behaviors to adapt to environmental changes in a certain time sequence. Mammalian biological clocks are divided into central biological clocks and peripheral biological clocks. The mammalian central circadian clock is in the suprachiasmatic nucleus (SCN), known as the central pacemaker of the circadian clock, and plays a role in maintaining the correct phase alignment of peripheral tissue clocks. The peripheral clock is distributed in tissues other than the SCN. As the central pacemaker of the biological clock, the SCN can regulate the synchronization of the peripheral clock with the central clock through autonomic innervation, body temperature, and hormone secretion (1). Entrainment mechanisms arise when the phase of a molecular clock is regulated by external environmental cues to synchronize with its time (2). As a peripheral tissue, the skeletal muscle circadian clock is entrained by external environmental cues. Entrainment cues (Zeitgebers) such as the light-dark cycle (3), feeding time, and skeletal muscle activity (4) can all produce an entrainment effect on its peripheral circadian clock to form an independent biological rhythm. This tissue-specific rhythm plays an important role in physiological functions such as energy metabolism and myogenesis (5).

Mammalian mitochondria are often referred to as the power source of cells. They are the main organelles for energy metabolism in the body and the main source of cellular ATP. They are also the most active parts and main site of the tricarboxylic acid cycle, respiration, and the synthesis of high-energy phosphate compounds in eukaryotic cells. Previous studies have shown that endurance exercise can induce mitochondrial biogenesis, leading to improvements in mitochondrial mass and respiratory function. Alternatively, acute heavy exercise can cause skeletal muscle mitochondrial swelling (6) and transient impairment of respiratory function (7) owing to metabolite accumulation in the skeletal muscle, and mitochondrial exposure to an environment of elevated lactate can lead to impaired respiratory function (8). The energy metabolism of mammals is closely related to the biological clock system. Under the regulation of the molecular clock, the process of energy metabolism exhibits a rhythmic oscillation pattern (9). A lack of *Clock* genes can lead to a series of metabolic disorder syndromes such as obesity and diabetes. Studies have shown that the clock gene brain and muscle ARNT-like 1 (*Bmal1*) plays an important role in energy metabolism (10), regulating skeletal muscle mitochondrial morphology, protein expression, and posttranslational modification processes in proteins, resulting in rhythmic changes in mitochondrial metabolic function. Liver proteomics have revealed that critical catabolic and oxidative functions of mitochondria exhibit circadian oscillations, and the recovery of skeletal muscle mitochondrial function impairment is associated with its dynamic circadian characteristics (11).

In the peripheral tissue, the transcription-translation feedback loop (TTFL) regulates mitochondrial deacetylation to rhythmically synchronize nicotinamide adenine dinucleotide (NAD^+^) biosynthesis with the circadian clock. Silent information regulator factor 2-related enzyme 1 (SIRT1) deacetylation silences circadian clock activity in Bmal1-regulated metabolic tissues (12). Nicotinamide phosphoribosyl transferase (NAMPT) transcription is directly regulated by the CLOCK: BMAL1 heterodimer through the E-box in its promoter (13). The biosynthesis of NAD^+^ is dependent on NAMPT, and SIRT1 activity is dependent on the control of NAD^+^ (14). Therefore, expression of the NAMPT/NAD^+^/SIRT1 signaling axis is regulated by the peripheral clock genes *Bmal1* and *Clock* (15), and NAMPT oscillates in circadian rhythm in skeletal muscle (16). In addition, experiments have shown that this signaling axis plays an important role in regulating skeletal muscle energy metabolism (17, 18). Peroxisome proliferator-activated receptor-γ coactivator-1α (PGC-1α) is rhythmically expressed in rat skeletal muscle, and exercise can induce mitochondrial biogenesis in skeletal muscle by upregulating PGC-1α. PGC-1α can also act as a target of SIRT1 to integrate the circadian clock and energy metabolism (19). Exercise disrupts NAD^+^ expression in skeletal muscle to control cellular redox capacity, and SIRT1 is a key regulator of acute and chronic exercise-mediated mitochondrial adaptation in skeletal muscle (20). In rodents, endurance exercise increases NAMPT expression and affects NAD^+^ levels, and the expression of SIRT1 has been shown to be increased after exercise (21). Therefore, it has been suggested that the NAMPT/NAD^+^/SIRT1 signaling pathway is a key hub for the BMAL1-dependent rhythmic oscillation of mitochondrial respiration (22), which is closely related to the regulation of energy metabolism by the biological clock. However, there are few studies focused on the peripheral biorhythm mechanisms of skeletal muscle energy metabolism disorder induced by heavy-load exercise.

The purpose of this research was first to verify that acute heavy-load exercise affects skeletal muscle peripheral circadian clock gene rhythm oscillations and energy metabolism processes. Second, we hypothesized that the potential key mechanism of peripheral circadian regulation of skeletal muscle energy metabolism by clock genes after heavy-load exercise is the NAMPT/NAD^+^/SIRT1 signaling pathway. We analyzed the energy metabolism-related changes in skeletal muscle in rats after acute heavy-load exercise to verify this research hypothesis. To do this, we analyzed the mitochondrial ultrastructure of rat skeletal muscle, respiratory chain function, clock gene expression, rhythmic oscillation and other effect changes, changes in the content or activity of signaling axis-related factors, and the interaction of skeletal muscle clock genes with this signaling pathway.

## MATERIALS AND METHODS

### Animal and Exercise Protocol

Eight-week-old SPF-grade, healthy, male Sprague–Dawley rats [weight: 200–230 g; License No. SCXK (Chuan) 2020-030] were purchased from Chengdu Dashuo Biotechnology Co., Ltd. The Ethics Committee of the Sports Science Experiments of Chengdu Sport University approved this study. Rats were fed in separate cages, with four animals per cage. All rats had free access to food and water. Rats were kept in an environment with a temperature of 20°C–26°C, a relative humidity of 40%–70%, and an alternating cycle of 12-h light and 12-h dark. Lights were turned on at 08:00 am, which was defined as zeitgeber time (ZT) 0; lights were turned off at ZT12 (20:00). After 3 days of adaptive feeding, rats were randomly divided into two groups. The control group (C) was subdivided into 13 subgroups (C0h, C6h, C12h, C18h, C24h, C30h, C36h, C42h, C48h, C54h, C60h, C66h, and C72h) based on different sampling time points (ZT0, ZT6, ZT12, ZT18, ZT24, ZT30, ZT36, ZT42, ZT48, ZT54, ZT60, ZT66, and ZT72, respectively). The exercise group (E) was also subdivided into 13 subgroups (E0h, E6h, E12h, E18h, E24h, E30h, E36h, E42h, E48h, E54h, E60h, E66h, and E72h) based on different sampling time points. According to the relevant literature (23) and our preliminary experiment, we determined that it was sufficient to have six rats in each group.

**Figure 1.**
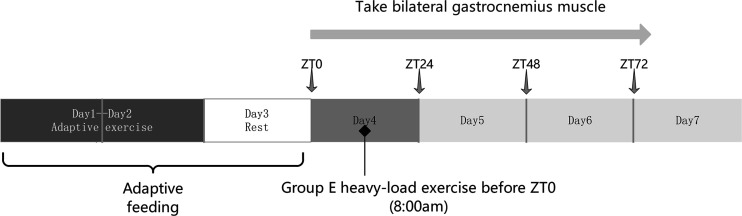
Schematic diagram of the experimental flow. ZT, zeitgeber time.

Before the initiation of formal treadmill exercise, all rats in *group E* underwent 2 days of adaptive training to acclimate them to the formal exercise protocol (24). The adaptive training involved a 0° incline, 16 m/min for 5 min on the first day; and a 0° incline, 16 m/min for 10 min on the second day. After 1 day of rest, the formal experiment was started. The rats in each exercise subgroup ran simultaneously on a motor-driven treadmill before the light start time ZT0; the rats ran continuously at an incline of −16° and at a speed of 16 m/min for 90 min (25). Rats were encouraged to run by the use of small bars at the ends of the treadmill lanes. The control group (C) was fed normally without exercise intervention. If the rats could complete the exercise regimen, they were included in the study. If a rat died or could not complete the exercise regimen, they were excluded. A total of 156 rats were eventually included in the study (Fig. 1).

### Sample Collection

After exercise, skeletal muscle samples were collected from the two groups of rats. According to the corresponding phase, we collected tissues under dim red light during the dark period (ZT12–ZT24) to minimize light cues. The rats were weighed and then anesthetized. The dose standard was 3.5 mL/kg body wt, 10% chloral hydrate was injected, the neck was severed, and the bilateral gastrocnemius muscles were quickly removed. The muscles were then rinsed with ice-cold physiological saline to remove blood, wiped dry with filter paper, connective tissue was removed, and the muscles were then segmented for later use. Of these, the middle tissue section of ∼2 mm × 3 mm was taken from the left gastrocnemius muscle and deposited in 2.5% glutaraldehyde fixative to prepare for electron microscopy. The remaining tissue was wrapped in tin foil and stored in liquid nitrogen, and then moved to an −80°C refrigerator for subsequent RT-qPCR and ELISA. The right gastrocnemius skeletal muscle was equally divided into three sections, one section was placed in a 4% paraformaldehyde solution and stored at 4°C for immunofluorescence colocalization analysis, the other section was used for Western blotting, and the remaining tissue was immediately placed in a centrifuge tube for mitochondrial extraction.

### RNA Isolation and Real-Time Quantitative PCR Analysis

Muscle tissues (50 mg) were ground in liquid nitrogen and then 1 mL of prechilled TRIzol was added; and the muscle tissues were centrifuged for 10 min (10,000 *g*, 2°C–8°C). Then, 75% ethanol precipitation was used to isolate RNA, followed by centrifugation for 3 min (2,400 *g*, 2°C–8°C). The supernatant was discarded and total RNA remained. The concentration and purity of RNA were analyzed by Ultra-micro UV spectrophotometer and agarose gel electrophoresis, and cDNA was synthesized by reverse transcription.

PIKORed 96 (produced by Thermo Fisher) was used to measure the expression levels of Bmal1 and Clock mRNA via real-time fluorescence quantitative PCR. The primers used to measure the expression were designed and synthesized by Shanghai Shengong Bioengineering Management Technology Development Service Co., Ltd., and purified by ULTRAPAGE. The primer sequence is shown in Table 1. Real-time quantitative PCR was performed and the CT (threshold cycle) values of each sample were analyzed using Thermo Scientific PikoReal software. The relative mRNA content level was calculated via 2^−ΔΔCT^.

**Table 1.** PCR primer sequences

Primer Name	Upstream	Downstream
β-Actin	5′-GAAGATCAAGATCATTGCTCC-3′	5′-TACTCCTGCTTGCTGATCCA-3′
*Bmal1*	5′-TGCCACCAATCCATACAC-3′	5′-TTCCCTCGGTCACATCCTAC-3′
*Clock*	5′-TGCTGGAAAGTGACTCCTTAACCC-3′	5′-TCATAGGTGGATGGCTCCTTTGGG-3′

### Transmission Electron Microscopy of Skeletal Muscle Mitochondria

Samples (50 mg) were prefixed with 3% glutaraldehyde at the beginning and end of each cycle (ZT0, ZT24, ZT48, and ZT72), then fixed with 1% osmium tetroxide and dehydrated with acetone; the concentration gradient of the dehydrating agent was 30%→50%→70%→80%→90%→95%→100% (three times for 100% concentration). Samples were then embedded with epoxy resin and ultrathin sections with a thickness of ∼50 nm were prepared using an ultramicrotome. The samples were first stained with uranyl acetate and then with uranyl acetate and lead citrate at room temperature for 15–20 min. Finally, gastrocnemius mitochondrial morphology was observed via JEM-1,400 Flash transmission electron microscopy (TEM) at ×15,000 magnification.

### Extraction of Skeletal Muscle Mitochondria

In this study, a mitochondrial separation kit (Biyuntian, C3606) was used to extract mitochondria from 80 to 100 mg of fresh tissue at the beginning and end of each cycle (ZT0, ZT24, ZT48, ZT72) using the differential centrifugation method. Following the kit instructions, we washed the tissues with PBS once and then they were cut up. The supernatant was obtained by multiple rounds of centrifugation at 600 *g* for 20 s, and then the mitochondrial separation reagent was added. Then, the sample was stroked 20–30 times with a handheld electric homogenizer (Shanghai FLUKO Co., F10 handheld homogenizer) on ice and centrifuged at 600 *g* and 4°C for 5 min. The supernatant was then carefully transferred to another centrifuge tube and centrifuged at 11,000 *g* and 4°C for 10 min. The supernatant was carefully removed and an appropriate amount of pregranulated mitochondrial storage solution was added. The sample was then immediately frozen in liquid nitrogen and stored at −80°C until use.

### Enzyme-Linked Immunosorbent Assay

#### Determination of NAMPT content.

The rat nicotinamide phosphoribosyl transferase (NAMPT) ELISA kit (Shanghai Enzyme-linked Biotechnology Co., Ltd., ml059081) was used to determine NAMPT content at the beginning and end of each cycle. Muscle tissue (30 mg) was weighed, 360 μL of PBS (pH 7.4) was added, and a homogenizer was used to fully homogenize the specimen. Samples were then centrifuged for 20 min (600 *g*), and the supernatant was collected. Standard wells contained 50 μL of the standard (two duplicate wells) and blank wells contained 50 μL diluent. For the samples, 40 μL of diluent was added to the sample wells and then a 10-μL sample was added. Then, 100 μL of the enzyme-labeled reagents were added to each well except the blank wells, and the plate was incubated at 37°C for 60 min after sealing with a sealing plate membrane. The plates were then uncovered, the liquid discarded, and the plates dried by swinging. A wash solution was diluted 20-fold with distilled water and reserve was added to every well and allowed to sit for 30 s before draining; this was repeated five times. The plates were then dried by patting and visualized. For visualization, chromogen solution A (50 μL) and chromogen solution B (50 μL) were added to each well, and the plates were gently shaken and allowed to sit for 15 min at 37°C in the dark. The reaction was stopped by adding the Stop Solution (50 μL) to each well and the plates were assayed. The blank well was taken as zero, and the absorbance (OD value) of each well was measured at a wavelength of 450 nm. The sample concentration was calculated using the standard curve and multiplication by the dilution factor of five.

#### Determination of NAD^+^ content.

NAD^+^ content was determined at the beginning and end of each cycle using an NAD^+^/NADH detection kit (Biyuntian, S0175). Tissues were washed with precooled PBS, 30 mg of tissue was weighed and cut with scissors, and 400 μL of NAD^+^/NADH extract was added. The samples were homogenized on ice, centrifuged at 12,000 *g* for 10 min at 4°C, and the supernatants removed. The NADH standard was prepared and 655 μL of NADH preparation solution and 5 mg NADH were used to obtain a 10 mM standard. The NADH standard curve was set and the NADH standard was diluted to create a 0 μM, 0.25 μM, 0.5 μM, 1 μM, 2 μM, 4 μM, 6 μM, 8 μM, and 10 μM concentration gradient. Determination of total NAD^+^ and NADH was achieved by pipetting 50 μL of the sample into a centrifuge tube and placing it in a water bath at 60°C to decompose the NAD^+^. Samples were then centrifuged at 10,000 *g* for 5 min at 4°C, and then 20 μL of supernatant was pipetted into a 96-well plate. The sample to be tested, NAD^+^/NADH extract, and alcohol dehydrogenase working solution were added and the plates were incubated at 37°C for 10 min in the dark. Then, 10 μL of chromogenic solution was added to each well and mixed before incubation at 37°C in the dark for 30 min. Absorbance at 450 nm was measured and results were calculated. The standard curve was drawn by taking the NADH concentration as the abscissa and the absorbance as the ordinate. The total concentration and NADH concentration in the sample were calculated based on the standard curve and then calculated using the formula: [NAD^+^] = [NAD_total]_ − [NADH] to obtain the NAD^+^ content.

#### Determination of SIRT1 activity.

SIRT1 activity was assayed at the beginning and end of each cycle using the SIRT1 Activity Fluorescence Assay Kit (Abcam, ab156065); all experimental steps were performed at 4°C. For the isolation of nuclei, tissue samples (100 mg) were minced and resuspended in 2 mL of isolation buffer containing 10 mM Tris·HCl (pH 7.5), 10 mM NaCl, 15 mM MgCl_2_, 250 mM sucrose, and 0.1 mM EGTA. Samples were homogenized and poured into a centrifuge tube, centrifuged at 800 *g* for 10 s, set in an ice bath for 15 min, and then centrifuged at 300 *g* for 10 min. The supernatant was removed, gently resuspended, and washed with prechilled 10 mM NaCl and 10 mM Tris·HCl (pH 7.5). The isolated nuclei were resuspended in 100 μL of extraction buffer containing 50 mM HEPES KOH (pH 7.5), 420 mM NaCl, 0.5 mM EDTA Na_2_, 0.1 mM EGTA, and 10% glycerol and sonicated for 30 s. Samples were then placed in an ice bath for 30 min, centrifuged at 20,000 *g* for 10 min and the supernatant removed for analysis. Protein concentration was determined using the bicinchoninic acid (BCA) protein concentration assay kit (Biyuntian, P0009) and then the samples were stored at −80°C for subsequent SIRT1 activity assays. Sirt1 deacetylase activity in the nuclear extracts was quantified according to the kit instructions. The fluorescence intensity was read for 30 to 60 min at 1- to 2-min intervals using a microtiter plate fluorometer with excitation at 340–360 nm and emission at 440–460 nm. The rate of the reaction was measured and calculated while the reaction velocity remained constant.

#### Determination of COX II/IV activity.

The rat respiratory chain complex II ELISA assay kit (Abcam, ab109908) and the respiratory chain complex IV ELISA assay kit (Abcam, ab109911) were used to measure the activity of the extracted mitochondrial respiratory chain complexes. The assays were carried out according to the kit instructions. Activity was determined using the following formula: rate (OD/min) = OD1 − OD2/time (min) = OD (*t*1 − *t*2)/△*t*. The activities of complex II (COX II) and complex IV (COX IV) were calculated in mOD/min. To exclude the influence of the experimental environment and operation errors on the mitochondrial complex activities, relative activity was used to represent the activity value of each group and each phase.

#### Determination of ATP/ADP content.

The rat ATP ELISA kit (Jiyinmei, JYM0725Ra) and rat ADP ELISA kit (Jiyinmei, JYM0724Ra) were used at the beginning and end of the cycle for ATP/ADP content determination. Tissue samples were cut, and 40 mg of tissue were frozen in liquid nitrogen and stored at −80°C for future use. The tissue samples were homogenized after adding PBS (pH 7.4); samples were kept at 4°C. The supernatant was collected after centrifugation for 20 min at 600 *g*. The supernatant was aliquoted for ELISA assays and stored for future use. The collected supernatant was divided into two portions and ATP and ADP were determined. Ten wells were set as standards in a Micro Elisa strip plate. In *well 1* and *well 2*, 100 μL of standard solution and 50 μL of standard dilution buffer were added and mixed well. In *well 3* and *well 4*, 100 μL of solution from *well 1* and *well 2* were added, respectively. Then, 50 μL of standard dilution buffer was added and mixed well, and 50 μL solution were discarded from *well 3* and *well 4*. In *well 5* and *well 6*, 50 μL of solution from *well 3* and *well 4* were added, respectively. Then, 50 μL of standard dilution buffer were added and mixed well. In *well 7* and *well 8*, 50 μL of solution from *well 5* and *well 6* were added, respectively. Then, 50 μL of standard dilution buffer were added and mixed well. In *well 9* and *well 10*, 50 μL of solution from *well 7* and *well 8* were added, respectively. Then, 50 μL of standard dilution buffer were added and mixed well, and 50 μL of solution were discarded from *well 9* and *well 10*. After dilution, the total volume in all the wells was 50 μL and the concentrations were 360 pg/mL, 240 pg/mL, 120 pg/mL, 60 pg/mL, and 30 pg/mL. In the micro-ELISA strip plate, one well was left empty as the blank control. In the sample wells, 40 μL of sample dilution buffer and 10 μL of sample were added (dilution factor was 5).

Samples were loaded onto the bottom without touching the well wall and mixed well with gentle shaking. Then, 50 μL of horseradish peroxidase (HRP)-conjugate reagent was added to each well except the blank control well, and plates were incubated for 30 min at 37°C after sealing with the closure plate membrane. The concentrated washing buffer was diluted with distilled water 30 times. Then, the closure plate membrane was peeled off, the liquid was aspirated, and the wells were refilled with the wash solution. The wash solution was discarded after resting for 30 s. The washing procedure was repeated five times. Chromogen solution A (50 μL) and chromogen solution B (50 μL) were added to each well, mixed with gentle shaking, and incubated at 37°C for 10 min in the dark. After incubation, 50 μL of stop solution was added to each well to terminate the reaction. Absorbance was read at an OD of 450 nm using a microtiter plate reader within 15 min of adding the stop solution. The OD value of the blank control well was set as zero, and the sample concentration was calculated using the standard curve and multiplication by the dilution factor of five.

### Western Blotting

Muscle samples (40 mg) from the beginning and end of each cycle were placed in tubes and 400 µL of Radio Immunoprecipitation Assay (RIPA) lysis buffer was added to each tube (Servicebio, China) (according to the mass ratio of the rat gastrocnemius muscle:lysis buffer = 1:10). The tissue was cut into pieces, lysed on ice for 10 min, put into a low temperature, low-speed grinding instrument (Servicebio, China) to be homogenized, and centrifuged at 4°C and 13,800 *g* for 10 min. Protein concentration was determined using a BCA protein concentration assay kit (Biyuntian; P0009). The protein concentration was leveled with the lysate according to the standard curve, and 50 μL of the leveled protein liquid were added to 5× loading buffer in a 4:1 ratio. After mixing was completed, the protein mixture was put into a thermal cycler (TECHNE TC-3000), 95°C, 15 min, for protein denaturation, and stored in the refrigerator at −20°C for later use. The Yaenzyme PAGE Gel Rapid Preparation Kit (Shanghai Yaenzyme; PG112) was used to make the gels (10%). After electrophoresis, samples were transferred to PVDF membranes, the membranes were blocked, and primary antibodies were added. The primary antibodies used were: Anti-bmal1 (Sigma Aldrich, Cat. No. SAB2500166, RRID:AB 10603425, diluted 1:1,000), anti-clock (Abcam, Cat. No. ab3517, RRID:AB 303866, diluted 1:2,000), anti-PGC-1α (Abcam, Cat. No. ab72230, RRID:AB 1640773, diluted 1:1,000), and anti-GAPDH (Abcam, Cat. No. ab8245, RRID:AB 2107448, diluted 1:100,000). Membranes were then shaken gently at 4°C and incubated overnight. Membranes were then washed with Tris-buffered saline-Tween 20 (TBST) three times for 5 min each time. Appropriate secondary antibodies were then added, and the membranes were incubated at room temperature with gentle shaking for 2–3 h. Then, TBST was used to wash the membrane three times for 10 min each time. Bands were then visualized using ECL luminescent solution and scanned using the Teneng GIS case-control software V2.0. Results are expressed as the relative expression level of the target protein.

### Immunofluorescence Colocalizition

The fixed tissue samples at the beginning and end of each cycle were dehydrated using an automatic dehydrator and then deparaffinized. The sections were put into xylene I for 15 min, xylene II for 15 min, xylene III for 15 min, anhydrous ethanol I for 5 min, anhydrous ethanol II for 5 min, 85% alcohol for 5 min, and 75% alcohol for 5 min. Sections were then washed with distilled water before antigen retrieval. For antigen retrieval, the slices were put into citrate buffer (pH 6.0), heated in a microwave for 10 min on high, rested for 8 min, and then heated for 10 min on medium-high heat. After being cooled, sections were washed three times with PBS for 5 min each time. Then, goat serum blocking solution was added and sections were allowed to stand for 20 min at room temperature. Primary antibodies were then added and incubated overnight at 4°C. Then, the sections were washed with PSB three times for 5 min each time. The secondary antibodies were then added and incubated at 37°C for 30 min. Sections were washed three times with PBS for 5 min each time. Then, DAPI was added and sections were incubated at room temperature for 10 min and washed three times with PBS for 5 min each time. An antifluorescence decay mounting medium cover sheet was then used.

### Statistical Analysis

All data are presented as the means ± SE. Data were processed using GraphPad Prism 8.0 statistical software. The normality test was performed on the obtained data, the Student’s *t* test was used for the comparison of normally distributed data from two groups of independent samples, the least-significant different (LSD) test was used when the variances were homogeneous, and the Welch’s correction test was used when the variances were unequal. A *P* < 0.05 was considered to indicate a significant difference.

The cosine analysis software CircaCompare (*R* package) was used to obtain the parameters of the fitted cosine curve. The fitted cosine function equation was *Y* = mesor + mmplitude × cos (time_radians − φ), where mesor is the baseline/median value, amplitude is the rhythmic oscillation amplitude, φ is the peak phase, and time_radians is the radian value corresponding to the time.

## RESULTS

### Changes in the mRNA Expression and Rhythm of the Skeletal Muscle Clock Genes *Bmal1* and *Clock* after Heavy-Load Exercise

#### Changes in the mRNA expression of the skeletal muscle clock genes Bmal1 and Clock after heavy-load exercise.

**Figure 2.**
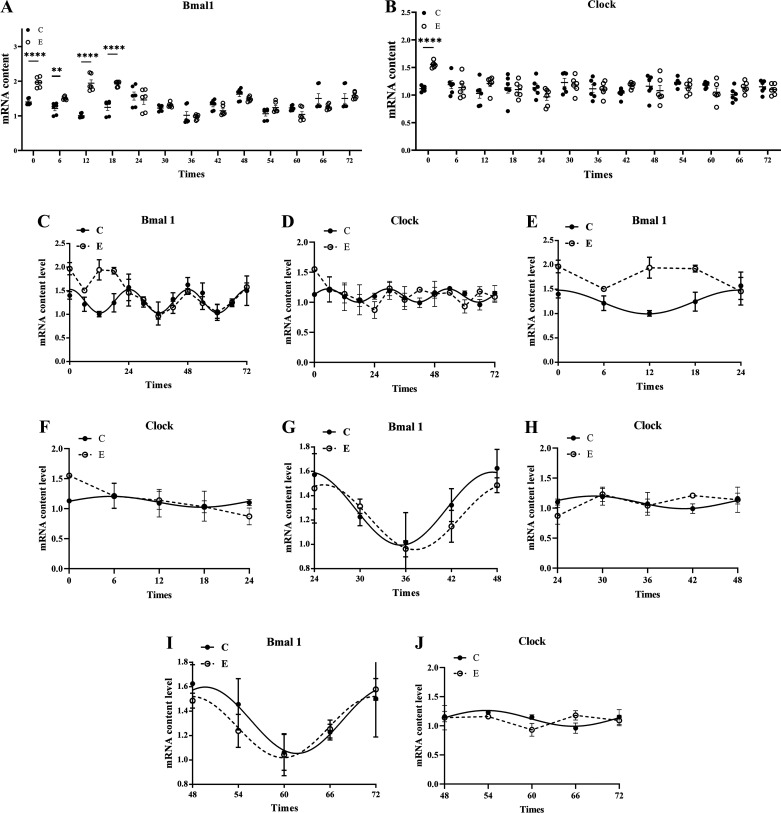
Changes in the mRNA expression and rhythm of the skeletal muscle clock genes *Bmal1* and *Clock* after heavy-load exercise. *Bmal1* mRNA expression graph: *Bmal1* mRNA expression changes within 72 h (*A*), *Clock* mRNA expression graph: *Clock* mRNA expression changes within 72 h (*B*), 0–72 h *Bmal1* rhythm oscillation graph: *Bmal1* mRNA rhythm changes within 72 h (*C*), 0–72 h *Clock* rhythm oscillation graph: *Clock* mRNA rhythm changes within 72 h (*D*), 0–24 h *Bmal1* rhythm oscillation graph: *Bmal1* mRNA rhythm changes from ZT0 to ZT24 (*E*), 0–24 h *Clock* rhythm oscillation graph: *Clock* mRNA rhythm changes from zeitgeber time (ZT)0 to ZT24 (*F*), 24–48 h *Bmal1* rhythm oscillation graph: *Bmal1* mRNA rhythm changes from ZT24 to ZT48 (*G*), 24–48 h *Clock* rhythm oscillation graph: *Clock* mRNA rhythm changes from ZT24 to ZT48 (*H*), 48–72 h *Bmal1* rhythm oscillation graph: *Bmal1* mRNA rhythm changes from ZT48 to ZT72 (*I*), 48–72 h *Clock* rhythm oscillation graph: *Clock* mRNA rhythm changes from ZT48 to ZT72 (*J*). Values are represented as means ± SE. **P < 0.01, ****P < 0.0001, *n* = 6/group.

To observe the effect of heavy-load exercise on clock genes, we detected the expression of the clock genes *Bmal1* and *Clock* mRNA in each subgroup by RT-qPCR. The results showed that compared with *group C*, the expression of Bmal1 mRNA in *group E* first increased and then decreased within 72 h after heavy-load exercise (Fig. 2*A*). Significant upregulation occurred at ZT0 (*P* < 0.001), ZT6 (*P* = 0.006), ZT12 (*P* < 0.001), and ZT18 (*P* < 0.001) (Fig. 2*A*). *Bmal1* mRNA in *group E* decreased gradually from ZT24, and there was no significant difference between *group E* and *group C* at ZT72 (*P* > 0.05). The change in the trend of *Clock* mRNA was like that of *Bmal1* mRNA (Fig. 2*B*), which was significantly increased in *group E* at ZT0 (*P* < 0.001), with no significant difference between *group E* and *group C* in other phases (*P* > 0.05) (Fig. 2*B*).

#### Changes in the rhythm of the skeletal muscle clock genes Bmal1 and Clock after heavy-load exercise.

To determine whether heavy-load exercise can change the biological rhythm of skeletal muscle *Bmal1* and *Clock*, we used cosine analysis software to process the above RT-qPCR detection results. Results showed that during the period from ZT0 to ZT24, *Bmal1* mRNA in *group C* reached a peak at ZT0.25, decreased to a trough at ZT12.25, and then returned to ZT24 to complete a cycle of rhythmic oscillation (*P* = 0.041). In the next 2 days, its changing trend was similar to that of ZT0–ZT24, with three complete rhythmic cycles within 72 h (*P* = 1.61 × 10^−5^) (Fig. 2*C*). During the period from ZT0 to ZT24, the *Bmal1* mRNA in *group E* did not express rhythmically (*P* > 0.05) (Fig. 2*E*), but the rhythmic expression was restored from ZT24 to ZT48 (*P* = 0.001) (Fig. 2*G*) and ZT48 to ZT72 (*P* = 0.017) (Fig. 2*I*). *Clock* mRNA in *group C* peaked at ZT5.43 and then decreased to a trough at ZT17.43, returning to ZT24 to complete a cycle of rhythmic oscillation (*P* = 0.037) (Fig. 2*F*), showing a total of three complete rhythmic cycles within 72 h (*P* = 2.96 × 10^−5^) (Fig. 2, *H* and *J*). The circadian rhythm of *Clock* mRNA in *group E* disappeared within 72 h (*P* > 0.05) (Fig. 2*D*).

### Changes in the Morphological Structure of Skeletal Muscle Mitochondria at the Beginning and End of Each Cycle after Heavy-Load Exercise

**Figure 3.**
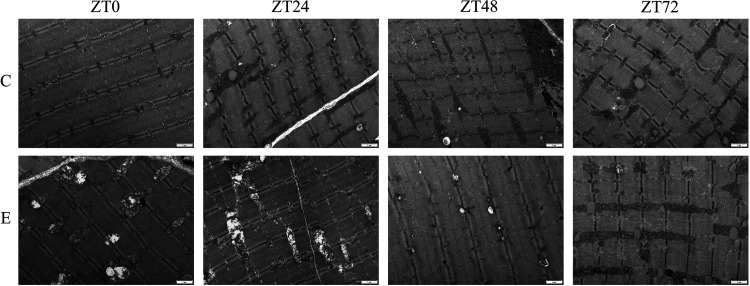
Electron micrographs of each group of skeletal muscle at the beginning and end of each circadian rhythm cycle. Changes of skeletal muscle mitochondrial morphology and structure at the beginning and end of each cycle in *groups C* and *E* after high-load exercise. ZT, zeitgeber time.

To observe the energy metabolism state of skeletal muscle at the beginning and end of each cycle after heavy-load exercise, we observed the ultrastructure of the gastrocnemius muscle mitochondria from the morphological point of view using a transmission electron microscope. The results showed that in *group C*, the Z line was smooth in each phase and the A-band and I-band were clearly demarcated. Mitochondria were evenly distributed on both sides of the Z line, long, linear or oval, with clear cristae structure, and neatly arranged and morphologically intact. At ZT0 in *group E*, the Z line was irregular, the mitochondria were unevenly distributed, irregular in shape, different in size, began to swell, and the cristae structure was unclear. At ZT24, the mitochondrial morphology had recovered somewhat, and the missing cristae structure began to appear. At ZT48, the shape of some mitochondria recovered, showing an elongated rod or oval shape, and mitochondrial damage was alleviated. At ZT72, the mitochondrial shape had essentially returned to normal (Fig. 3).

### Changes in Protein Expression of Skeletal Muscle BMAL1, CLOCK, and PGC-1α at the Beginning and End of Each Cycle after Heavy-Load Exercise

**Figure 4.**
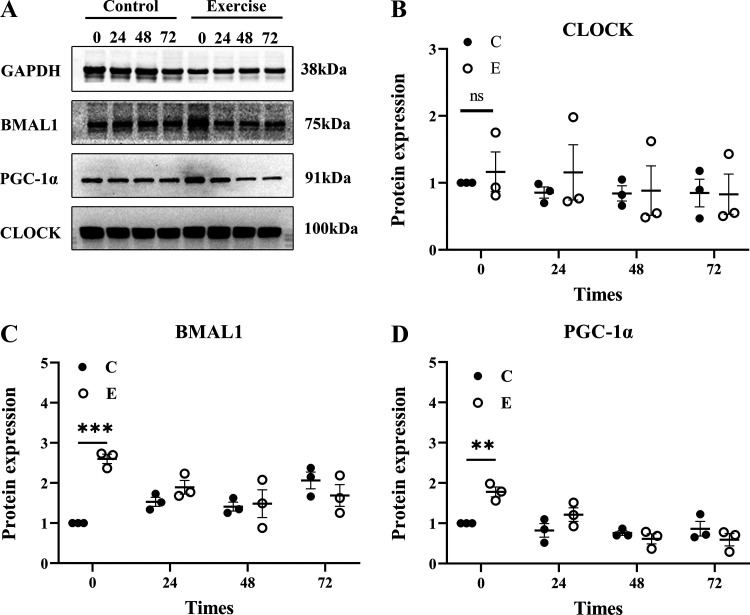
Changes of skeletal muscle clock genes and energy metabolism-related protein expressions at the beginning and end of each cycle after heavy-load exercise. Western blot bands: Western blot of *Clock* gene brain and muscle ARNT-like 1 (BMAL1), Clock, and peroxisome proliferator activated receptor γ coactivator-1α (PGC-1α) proteins (*A*); protein expression graph: changes in protein expression of skeletal muscle CLOCK, BMAL1, and PGC-1α at the beginning and end of each cycle after heavy-load exercise (*B*–*D*). Values are represented as means ± SE. **P < 0.01, ***P < 0.001, *n* = 6/group.

To evaluate the changes in clock genes and energy metabolism regulatory proteins at the beginning and end of each cycle after heavy exercise, we used Western blotting to detect the protein expression levels of BMAL1, CLOCK, and PGC-1α (Fig. 4*A*). The results showed that compared with *group C*, the expression BMAL1 in *group E* was significantly increased at ZT0 (*P* = 0.005) and then gradually decreased; there was no significant difference compared with *group C* at ZT24, ZT48, and ZT72 (*P* > 0.05) (Fig. 4*C*). There was also no significant difference in CLOCK protein expression between *groups C* and *E* at ZT0, ZT24, ZT48, and ZT72 (*P* > 0.05) (Fig. 4*B*). Compared with *group C*, PGC-1α in *group E* was significantly increased at ZT0 (*P* = 0.003), where at ZT24, ZT48, and ZT72, there was no significant difference compared with *group C* (*P* > 0.05) (Fig. 4*D*).

### Disturbance of Skeletal Muscle Energy Metabolism Induced by Heavy-Load Exercise

#### Changes in the NAMPT/NAD^+^/SIRT1 signal pathway at the beginning and end of each cycle after heavy-load exercise.

**Figure 5.**
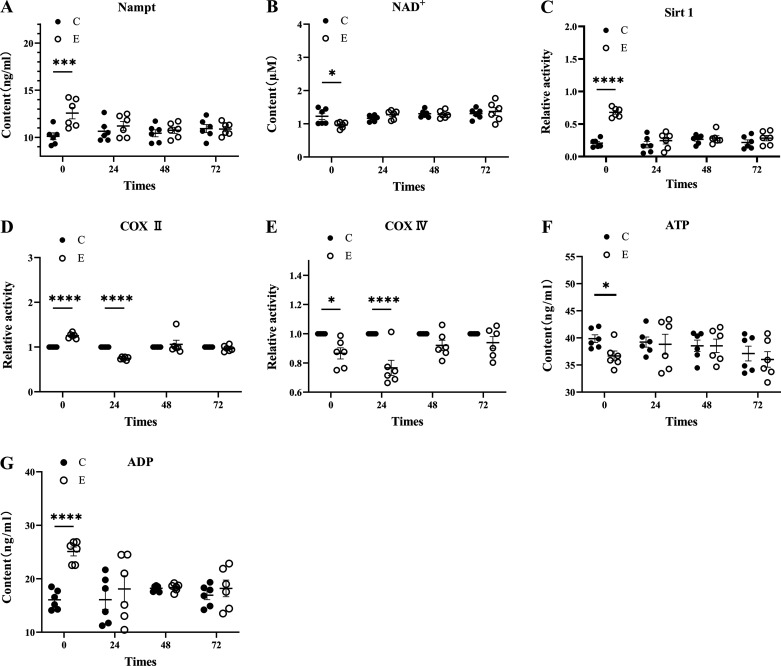
Changes in skeletal muscle energy metabolism disorder and nicotinamide phosphoribosyl transferase (NAMPT)/nicotinamide adenine dinucleotide (NAD^+^)/silent information regulator factor 2-related enzyme 1 (SIRT1) signaling axis after heavy-load exercise. *A*: Nampt content graph: changes in the NAMPT content at the beginning and end of each cycle after heavy-load exercise in each group. *B*: NAD^+^ content graph: changes in the NAD^+^ content at the beginning and end of each cycle after heavy-load exercise in each group. *C*: Sirt1 activity graph: changes in the Sirt1 activity at the beginning and end of each cycle after heavy-load exercise in each group. *D* and *E*: complex (COX) II and COX IV activity graphs: changes in COX II and COX IV activity at the beginning and end of each cycle after heavy-load exercise in each group. *F* and *G*: ATP and ADP content graph: changes in ATP and ADP content at the beginning and end of each cycle after heavy-load exercise. Values are represented as means ± SE. *P < 0.05, ***P < 0.001, ****P < 0.0001, *n* = 6/group.

To further explore the relationship between skeletal muscle clock genes and energy metabolism, we measured the content and activity of the NAMPT/NAD^+^/SIRT1 signaling pathway by ELISA. Compared with *group C*, the content of NAMPT in *group E* was significantly increased at ZT0 (*P* = 0.008) and then gradually recovered, with no significant difference compared with *group C* (*P* > 0.05) (Fig. 5*A*). The content of NAD^+^ in *group E* was significantly lower than in *group C* at ZT0 (*P* = 0.041) (Fig. 5*B*). SIRT1 activity in *group E* was significantly increased at ZT0 (*P* < 0.001) and there was no significant difference in this signaling pathway between the two groups at ZT24, ZT48, and ZT72 (*P* > 0.05) (Fig. 5*C*).

#### Changes in COX II, COX IV, ATP, and ADP at the beginning and end of each cycle after heavy-load exercise.

To evaluate the energy metabolism level of skeletal muscle after heavy-load exercise, we detected the activity of the mitochondrial respiratory chain complex and the content of ATP and ADP. The results showed that compared with *group C*, the relative activity of COXII in *group E* was significantly increased at ZT0 (*P* < 0.001) and significantly decreased to the lowest activity at ZT24 (*P* < 0.001). There was no significant difference compared with *group C* at ZT48 and ZT72 (*P* > 0.05) (Fig. 5*D*). The relative activity of COX IV in *group E* was significantly decreased in *group C* at ZT0 (*P* = 0.017) and ZT24 (*P* = 0.006) and there was no significant difference compared with *group C* at ZT48 and ZT72 (*P* > 0.05) (Fig. 5*E*).

Compared with *group C*, the ATP content of *group E* decreased significantly at ZT0 (*P* = 0.012), and there was no significant difference compared with *group C* at ZT24, ZT48, and ZT72 (*P* > 0.05) (Fig. 5*F*). The ADP content of *group E* was significantly increased at ZT0 (*P* = 0.030) and there was no significant difference from *group C* at ZT24, ZT48, and ZT72 (*P* > 0.05) (Fig. 5*G*).

### Mechanisms of Skeletal Muscle Clock Gene Regulation of Energy Metabolism after Heavy-Load Exercise

**Figure 6.**
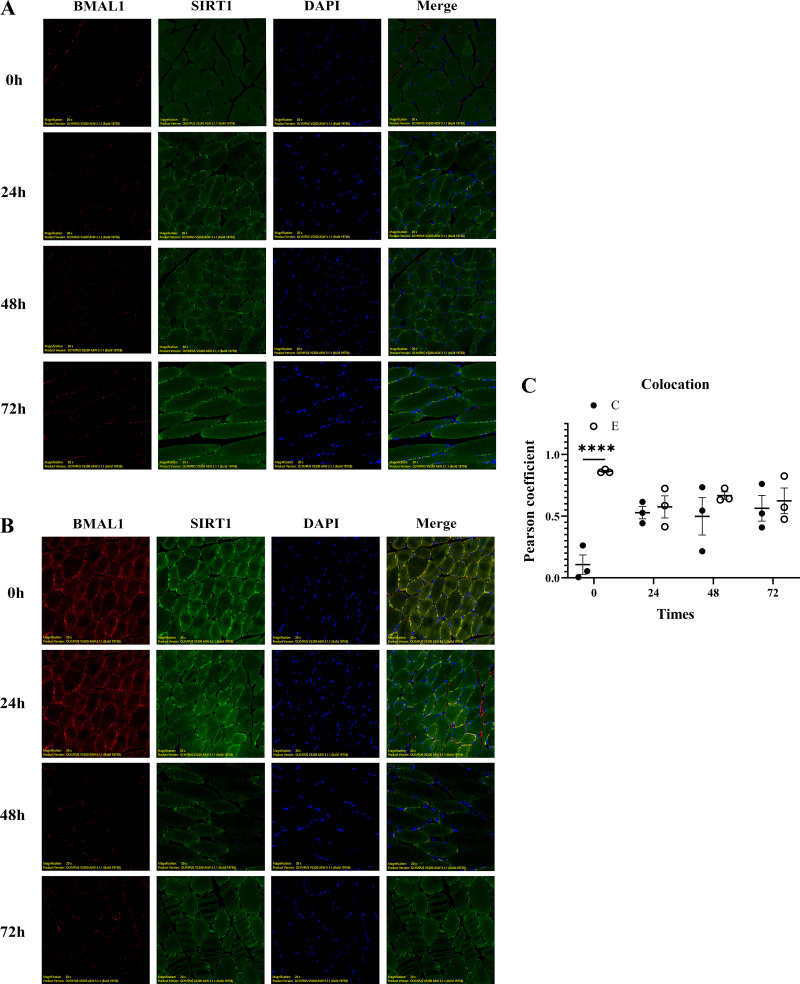
Colocalization of the core clock gene BMAL1 and the key molecule silent information regulator factor 2-related enzyme 1 (SIRT1) in the nicotinamide phosphoribosyl transferase (NAMPT)/nicotinamide adenine dinucleotide (NAD^+^)/SIRT1 signaling axis, BMAL1 is marked in red, SIRT1 is marked in green, nuclei were counterstained with DAPI, and colocalization is shown as yellow fluorescence. *Group C* BMAL1 and SIRT1 colocalization image: immunofluorescence colocalization of BMAL1 and SIRT1 in group (*A*); *group E* BMAL1 and SIRT1 colocalization image: immunofluorescence colocalization of BMAL1 and SIRT1 in *group E* (*B*); colocation index graph: Pearson correlation coefficient of immunofluorescence colocalization in *group C* and *group E* (*C*). Values are represented as means ± SE. ****P < 0.0001.

To verify the mechanisms of clock gene regulation of skeletal muscle energy metabolism after heavy exercise, we used immunofluorescence colocalization to observe the colocalization of the core clock gene BMAL1 and the key molecule SIRT1 in the NAMPT/NAD^+^/SIRT1 signaling axis. The results showed that there was no coexpression at ZT0, ZT24, ZT48, and ZT72 in *group C* (*P* > 0.05) (Fig. 6*A*). *Group E* showed colocalization at ZT0 (*P* < 0.001) and a small amount of colocalization markers remained at ZT24 (*P* > 0.05). The coexpression signal of ZT48 and ZT72 disappeared and there was no obvious marker (*P* > 0.05) (Fig. 6, *B* and *C*).

## DISCUSSION

In this research, rat skeletal muscle clock genes oscillated rhythmically. As a peripheral tissue, skeletal muscle has tissue-specific rhythms. *Bmal1* and *Clock* are rhythmically expressed as core clock genes in skeletal muscle (26). Previous studies have confirmed that the mRNA content of the core clock genes *Bmal1* and *Clock* in rat skeletal muscle oscillates in a circadian rhythm (27, 28), and the expression characteristics of rat skeletal muscle clock gene mRNA in this experiment were consistent with these studies. This result further demonstrates the critical role of skeletal muscle as a peripheral clock, indicating that the core clock genes *Bmal1* and *Clock* expressed in skeletal muscle are involved in maintaining the stable output of circadian rhythms.

In our research, skeletal muscle clock gene expression and rhythm were altered after heavy-load exercise and recovered gradually over time. As a zeitgeber, exercise can interact with clock genes, and studies have found that changes in jet lag across time zones can affect exercise performance (29). Under normal conditions of the central clock genes, exercise type, intensity, time, and exercise period can be used as external input signals to cause a phase shift of skeletal muscle core clock genes, resulting in disordered clock gene expression (4, 22). In this research, the mRNA and protein expression levels of the skeletal muscle clock genes *Bmal1* and *Clock* after heavy-load exercise were measured, and cosine function software was used to obtain and fit cosine curve parameters to analyze their changing trends. It was found that *Bmal1* mRNA in *group E* lost its oscillatory rhythm on the first day after exercise (ZT0–ZT24), and its protein expression was significantly increased at ZT0. The circadian rhythm of *Clock* mRNA disappeared within 72 h, but the protein expression did not change significantly, which proved that acute exercise caused abnormal expression and rhythm of the rat skeletal muscle clock genes. This is like previous studies showing that the rhythm of *Bmal1* and *Clock* expression in the skeletal muscle of mice was attenuated after acute exercise (24, 30). Human experiments have reported that the expression of *Bmal1* mRNA in the vastus lateralis muscle was significantly upregulated at 6 h and 18 h after a 30- to 45-min high-load resistance exercise in the lower limbs of the subjects, and the circadian rhythm was phase shifted (31). It is suggested that a heavy-load acute exercise can affect *Bmal1* and *Clock* mRNA and protein expression and oscillation rhythm, but their performance characteristics are different due to different molecular clocks. This may be because skeletal muscle is the main energy metabolism organ during exercise, and many genes involved in regulating energy metabolism have the characteristics of a circadian rhythm. It has been reported in the literature that 3.4% to 16% of genes in skeletal muscle are expressed in a rhythmic manner, and energy metabolism and clock genes can interact with each other, resulting in rhythm disorders and expression changes of clock genes in skeletal muscle (32, 33). Therefore, combined with the aforementioned results, we believe that the clock gene expression and rhythm disturbances in this study were induced by acute exercise, but the specific mechanism requires further exploration.

Mitochondria are the main sites of energy metabolism, providing energy for the activities of cells, and their structure and functional status determine the efficiency of energy supply. Changes in mitochondrial structure are mainly due to the changes in shape and size caused by fission, fusion, and autophagy. The mitochondrial respiratory chain located on the inner mitochondrial membrane provides most of the energy needed for cell survival, and its activity and ATP and ADP contents are important indicators of mitochondrial oxidative synthesis function. The structure and function of mitochondria are regulated by a variety of functional proteins. In recent years, PGC-1α has been shown to be a transcriptional coactivator that regulates energy metabolism and is the most important regulator of the mitochondrial quality control system. PGC-1α-deficient mice have impaired mitochondrial function in multiple tissues and are defective in adaptive responses to metabolic stress (34).

Studies have shown that changes in skeletal muscle energy metabolism-related gene expression may be affected by both exercise and postexercise circadian rhythms (35). First, unhabitual heavy-load exercise, especially eccentric exercise, can induce ultrastructural changes in skeletal muscle mitochondria (36). In this study, the exercise intervention in *group E* was a heavy-load downhill running exercise, and the gastrocnemius muscle performed eccentric contraction, which could cause changes in the ultrastructure of skeletal muscle mitochondria. COXII and COXIV are marker enzymes that reflect mitochondrial function. Their activity is enhanced, the efficiency of electron transfer in the respiratory chain is improved, and the oxygen utilization capacity of the tissue is enhanced, which is beneficial to tissue aerobic metabolism, marking an increase in intracellular energy ATP synthesis. Some experiments have also demonstrated that after subjects performed acute high-intensity exercise, the ability of the mitochondria in the vastus lateralis to produce ATP did not decrease immediately, but only began during the recovery period. An increase in the duration of exercise may have increased the demand for energy supply, thereby increasing the activity of intermediate metabolites, which is a kind of compensatory change (37, 38). During an acute high-intensity exercise, the skeletal muscle contracts violently, resulting in an immediate and significant change in COXIV activity and ATP and ADP content (39, 40). This experiment once again confirmed the instant effect of heavy-load exercise on mitochondrial oxidative respiration in skeletal muscle. In addition, studies have shown that clock genes play an important role in the maintenance of mitochondrial structure and function. Some mRNAs in mitochondria are periodically expressed, which can affect the dynamic stability of intracellular oxidative stress levels. Respiration of mitochondria shows rhythmic changes. When the circadian rhythm is disturbed, skeletal muscle can experience decreased mitochondrial volume and oxidative respiration, fiber-type translocation, altered sarcomere structure, and impaired muscle function (25, 41, 42). Some studies suggest that *Bmal1* may play a central role in the process of mitochondrial morphological changes, and many genes regulating mitochondrial morphology often rely on *Bmal1* to express rhythm (43). The gastrocnemius mitochondria of *Bmal1*^−/−^ mice showed morphological changes such as volume reduction or deletion, swelling, and cristae rupture, and the expression and rhythm of PGC-1α were significantly decreased (11). In addition, mitochondria in *Bmal1*^−/−^ mice are more susceptible to oxidative stress-related damage. Therefore, *Bmal1* deletion also makes mitochondria functionally abnormal (44). There are multiple transporters on the inner mitochondrial membrane, which are essential for the transport of metabolites. Mitochondrial proteins involved in many metabolic pathways are significantly affected by Clock-regulated acetylation, including the tricarboxylic acid cycle and proteins in glutathione metabolism, which depend on *Clock* acetylation sites (45). The clock genes can interact with the mitochondrial carrier protein SLC25A10 to regulate mitochondrial metabolism, thereby affecting the level of intracellular reactive oxygen species (ROS) (46). The results of this experiment are consistent with the changes in mitochondrial structure and function at the beginning and end of each cycle, as well as the rhythm of and changes in protein expression of *Bmal1*. This suggests that mitochondrial structure and function are regulated by clock genes, and *Bmal1* may be in a dominant position. Therefore, we speculate that the changes in mitochondrial structure and function in this study may be jointly regulated by exercise and circadian rhythm, but the pathway through which exercise and circadian rhythm play a regulatory role requires further exploration.

Cellular experiments have confirmed that the interaction between the circadian gene machinery and the NAMPT/NAD^+^/SIRT1 signaling axis is a key hub in regulating circadian oscillations in energy metabolism (22). BMAL1:CLOCK heterodimers can bind to E-BOX elements on downstream gene promoters and regulate their circadian expression in a time-dependent manner. NAMPT, an enzyme required for the biosynthesis of SIRT1’s essential cofactor NAD^+^ that determines the activity and rhythm of NAD^+^ and SIRT1, is targeted by BMAL1:CLOCK heterodimers (13, 15). BMAL1:CLOCK heterodimers can increase NAMPT expression, which in turn leads to an increase in NAD^+^, thereby increasing SIRT1 activity, which can regulate PGC-1α levels. It has thus been proposed that PGC-1α is rhythmically expressed under the control of clock genes and is a key component of the circadian oscillator that integrates the circadian clock and energy metabolism (47–49). In this study, we measured the molecular content and activity of the NAMPT/NAD^+^/SIRT1 signaling pathway at the beginning and end of each cycle, the expression of PGC-1α protein, the immunofluorescence colocalization of the clock gene *Bmal1*, and the key molecule SIRT1 in this signaling pathway. The purpose was to explore the possible regulatory mechanism of clock genes on the structure and function of skeletal muscle mitochondria after heavy-load exercise.

In our study, the molecular content and activity of the NAMPT/NAD^+^/SIRT1 signaling pathway and PGC-1α protein expression were significantly changed at ZT0 after exercise and gradually recovered in the following 2 days. Aerobic exercise can cause changes in skeletal muscle *Nampt* gene expression (50), and a single acute exercise can induce changes in *Nampt* gene expression. The results of this experiment showed that NAMPT content was significantly increased at ZT0 as in previous studies (51, 52). In this study, expression of the BMAL1 protein was significantly increased at ZT0, and the generation of NAMPT was positively regulated by BMAL1. Therefore, the key regulatory factor of the increase in NAMPT after a single bout of acute exercise may be the clock gene *Bmal1*. The literature shows that NAD^+^ levels in blood and muscle tissue are increased after moderate exercise and decreased after high-intensity exercise, and SIRT1 can be activated to increase its activity (53, 54). In this experiment, *group E* performed an acute heavy-load exercise. At ZT0, the content of NAD^+^ in skeletal muscle was significantly decreased immediately after exercise, the activity of SIRT1 was increased, and expression of the PGC-1α protein was upregulated. Therefore, the decrease in NAD^+^ content may have been caused by the exercise itself and SIRT1 activation. The colocalization of BMAL1 and SIRT1 was significantly upregulated at ZT0 after heavy exercise. This suggests that exercise can enhance the interaction between the BMAL1 and SIRT1 proteins, and SIRT1 can regulate PGC-1α, a key factor in mitochondrial biogenesis. The diurnal cycle changes of the aforementioned indices were consistent with the trend of *Bmal1* gene expression and rhythm change. Studies have confirmed that *Bmal1* is a nonredundant clock gene, the knockout of which can lead to complete disturbance of the biological clock (55). Therefore, we speculate that the development and outcome of skeletal muscle mitochondrial structure and function changes after heavy-load exercise may be mainly regulated by the clock gene *Bmal1*, potentially through the NAMPT/NAD^+^/SIRT1 signaling pathway. The immediate changes of this signaling pathway and PGC-1α after exercise may be mainly induced by acute exercise itself, and the regulatory effect of clock genes appear on the second day after exercise.

Although we did not separate the intrinsic cellular clock from timing factors (such as light/dark) in this study, we demonstrated in vivo the existence of a tissue-specific rhythm in rat peripheral skeletal muscle. In addition, the skeletal muscle circadian clock gene *Bmal1* may regulate the changes in the structure and function of skeletal muscle mitochondria caused by exercise, likely through the NAMPT/NAD^+^/SIRT1 signaling pathway. This theoretical speculation requires further research and verification. At present, research on the relationship between exercise and skeletal muscle energy metabolism has been extensive, but little is known about the peripheral biological rhythm mechanism. Understanding this mechanism is of great significance for exploring the regulation of mitochondrial structure and function by clock genes after overtraining in physical exercise. Furthermore, these results are important as they provide a theoretical basis for the timing of selected exercise; improving exercise performance; guiding weight loss exercise regimens; preventing and treating jet lag, shift discomfort, and depression; elevating the physical state of the military; and scientifically regulating an athlete's excitability during sports competition.

## DATA AVAILABILITY

Data will be made available upon reasonable request.

## GRANTS

This work was supported by the National Natural Science Foundation of China under Grant No. 81904318, Sichuan Natural Science Foundation under Grant No. 2023NSFSC0545, Sichuan Province Central Government Guides Local Science and Technology Development Project under Grant No. 2022ZYD0062, and Key Laboratory of Sports Medicine of the General Administration of Sport of China under Grant No. 2018YFF0300904. Y. Xia was supported by Sichuan Province Science and Technology Innovation and Entrepreneurship Seedling Project under Grant No. 2023JDRC0100 and the Sports Medicine Key Laboratory Project of Sichuan Province under Grant No. GS21ZX01.

## DISCLOSURES

No conflicts of interest, financial or otherwise, are declared by the authors.

## AUTHOR CONTRIBUTIONS

Y.X. conceived and designed research; Y.X., B.Y., Z.F., L.L., S.J., B.Q., Y.H., and H.D. performed experiments; Z.F. and L.L. collected and analyzed data; S.J., B.Q., and Y.H. performed experiments; Y.X., B.Y., and Z.F. interpreted results of experiments; B.Y. and Z.F. prepared figures; Y.X. and B.Y. drafted manuscript; Y.X. and B.Y. edited and revised manuscript; H.D. approved final version of manuscript.
